# Supplementary material for: Genetic Characterization of Kazakhstan Isolates: Avian Influenza H9N2 Viruses Demonstrate Their Potential to Infect Mammals
Source: Viruses. 2025 May 8;17(5):685. doi: 10.3390/v17050685 (PMC12115836; doi:10.3390/v17050685)
Supplement: Supplementary file 1 [file viruses-17-00685-s001.zip › viruses-3589732-supplementary.pdf]

Supplementary information

Genetic characterisation of Kazakhstan isolates of Avian Influenza H9N2 viruses demonstrate their potential to infect mammals

Contents

Supplementary Table S1. Molecular markers associated with the adaptation of H5 AIVs to mammalian hosts 1

Supplementary Table S2. Molecular characteristics of the neuraminidase amino acid sequences in the H9N2 AIV analysed in this study 4

Supplementary Figure S1. Phylogenetic tree of the PB2 gene full-length sequence of the H9N2 isolates from Kazakhstan. 5

Supplementary Figure S2. Phylogenetic tree of the PB1 gene full-length sequence of the H9N2 isolates from Kazakhstan. 6

Supplementary Figure S3. Phylogenetic tree of the PA gene full-length sequence of the H9N2 isolates from Kazakhstan. 7

Supplementary Figure S4. Phylogenetic tree of the NP gene full-length sequence of the H9N2 isolates from Kazakhstan. 8

Supplementary Figure S5. Phylogenetic tree of the M gene full-length sequence of the H9N2 isolates from Kazakhstan. 9

Supplementary Figure S6. Phylogenetic tree of the NP gene full-length sequence of the H9N2 isolates from Kazakhstan. 10

Supplementary Table S1. Molecular markers associated with the adaptation of H5 AIVs to mammalian hosts

Protein	Amino acid*			Viruses Tested in this study				Phenotype
	Residue	Avian-like motif	Mammalian-like motif	Pt/NK/6368	Ml/NK/6369	Ws/Sb/7994	Ck/Ay/220	
PB2	318	K	**R**	**R**	**R**	**R**	**R**	Increased polymerase activity in mice, mammalian cell lines [12]
	389	K	**R**	**R**	**R**	**R**	K	Increased polymerase activity and replication in mammalian cell line [24]
	504	I	**V**	**V**	**V**	**V**	**V**	Increased virulence in mice [12]
	588	A	**V**	A	**V**	A	**V**	

	598	V	**T**	**T**	V	**T**	V	Increased polymerase activity and replication in mammalian cells, increased virulence in mice [12, 24-26]
	627	E	**V**	E	**V**	E	**V**	
	89, 309	L, G	**V, D**	**V, D**	**V, D**	**V, D**	**V, D**	
	340, 588	R, A	**K, V**	**K,** A	**K, V**	**K,** A	R, **V**	Transmission in guinea pigs [4]
	89, 309, 339, 477, 495, 627, 676	L, G, T, R, I, E, A	**V, D, K, G, V, V, T**	**V, D, K, G, V,** E, **T**	**V, D, K, G, V, V, T**	**V, D, K, G, V,** E, **T**	**V, D, K, G, V, V,** M	Enhanced polymerase activity and increased virulence in mice [26]
PB1	3	D	**V**	**V**	**V**	**V**	**V**	Increased polymerase activity in mammalian cell line [4]
	13	L	**P**	**P**	**P**	**P**	**P**	Enhanced virulence in mammal's species [27]
	368	I	**V**	I	I	I	**V**	Playing a vital role for the adaptation of avian influenza viruses to a new host species [12]
	622	D	**G**	**G**	**G**	**G**	**G**	Increased polymerase activity and enhanced replication in mammalian cell line, increased virulence in mice [4]
PA	26	K	**E**	**E**	**E**	**E**	**E**	Increased polymerase activity in ferret [28]
	37	S	**A**	**A**	**A**	**A**	S	Increased polymerase activity in mammalian cell line [28]
	160	V	**D**	**D**	**D**	**D**	**D**	Increased polymerase activity in mice [28]
	63	V	**I**	V	V	V	**I**	Increased polymerase activity and enhanced replication in mammalian cell line, increased virulence in mice [4]
	356	K	**R**	K	K	K	**R**	
	383	N	**D**	**D**	**D**	**D**	**D**	Increased polymerase activity and replication in mammalian cell line [4, 28]
	409	N	**S**	**S**	**S**	**S**	N	
	436			E	E	E	E	Playing a vital role for the adaptation of viruses to a new host species [23]
HA	158	S	**N**	S	S	S	**N**	Increased virus binding to α2-6SA [26]
	183	K	**S**	N	N	N	**S**	Swine, mice; increased virus binding to α2–6, enhanced replication in mammalian cells [4]
	198	N	**T**	I	I	I	**T**	Increase replication and transmission in ferrets [4]

NP	136			L	L	L	L	Playing a vital role for the adaptation of avian influenza viruses to a new host species [12]
	398	K	**Q**	**Q**	**Q**	**Q**	**Q**	Increased polymerase activity in chicken, mice [23]
NA	30	A	**T**	A	A	A	**T**	Increased polymerase activity in ferret [25]
M1	15	V	**I**	V	V	V	**I**	Bat, chicken, mammalian cell lines [12]
	30	N	**D**	**D**	**D**	**D**	**D**	Increased virulence in mice [3, 4]
	215	T	**A**	**A**	**A**	**A**	**A**	
	166	A	**V**	**V**	**V**	**V**	A	
	43	I	**M**	**M**	**M**	**M**	**M**	Increased virulence in mice, chickens, and ducks [4]
NS1	42	P	**S**	**S**	**S**	**S**	**S**	Increased virulence in mice [4]
	103, 106	L, I	**F, M**	**F, M**	**F, M**	**F, M**	L, I	
	55, 66, 138	K, K, C	**E, E, F**	**E, E, F**	**E, E, F**	**E, E, F**	R, **E, F**	Enhanced replication in mammalian cells, decreased interferon response [4, 30]
	149	V	**A**	**A**	**A**	**A**	**A**	Increased virulence and decreased interferon response [30]
	227	E	**K**	E	E	E	**K**	Increased virulence in chicken and mice [4]

*A, alanine; C, cysteine; D, asparticacid; G, glycine; E, glutamicacid; F, histidine; I, isoleucine; K, lysine; L, leucine; M, methionine; N, asparagine; P, proline; Q, glutamine; R, arginine; S, serine; T, threonine; V, valine.

Supplementary Table S2. Molecular characteristics of the neuraminidase amino acid sequences in the H9N2 AIV analysed in this study

Strains	Hemadsorbing sites			Stalk deletions (TEI)	Antigenic determinants					Active center
	366-373 IRNGSRSG	399-403 DSENW	431-433 PQE		328-336	339-347	367-370	400-403	431-434	140-157
Pt/NK/6368/14	-	-	+	-	NDDSSSNSN	DPNNERGNP	SKDS	NNNW	PQET	LDNKHSNGTIHDRIPHRT
Ml/NK/6369/14	-	-	+	-	NDDSSSNSN	DPNNERGNP	SKDS	NNNW	PQET	LDNKHSNGTIHDRIPHRT
Ws/Ay/7994/19	-	-	+	-	NDDSSSNSN	DPNNERGNP	SKDS	NNNW	PQET	LDNKHSNGTIHDRIPHRT
Ck/Ay/220/20	-	-	+	+	NDDSSSSSN	DPNNERGAP	KTDS	CDDW	PQEP	LENKHSNGTTHDRTPHRT

*A, alanine; C, cysteine; D, asparticacid; G, glycine; E, glutamicacid; F, phenylalanine; H, histidine; I, isoleucine; K, lysine; L, leucine; M, methionine; N, asparagine; P, proline; Q, glutamine; R, arginine; S, serine; T, threonine; V, valine; W, tryptophan -, none

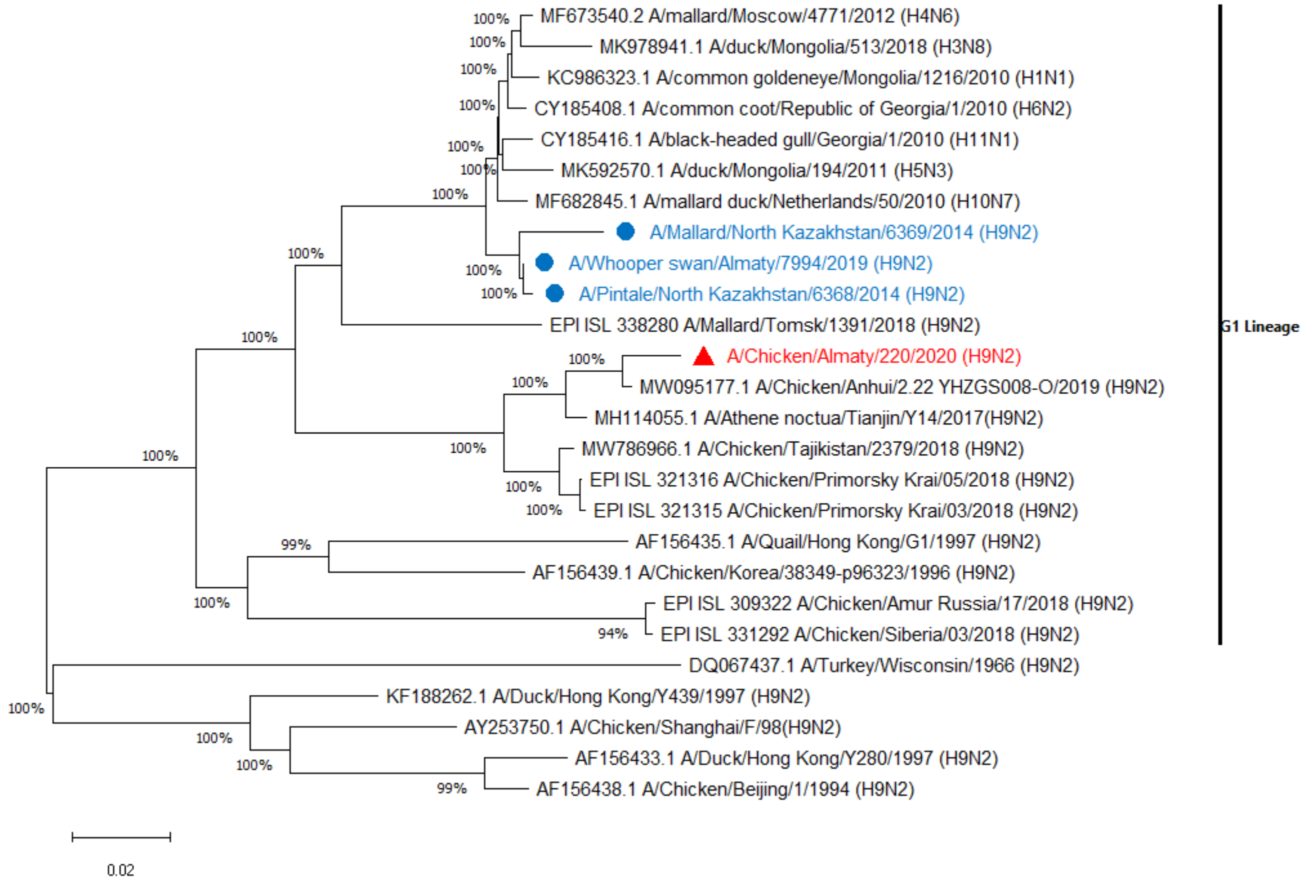

PB2 gene

Supplementary Figure S1. Phylogenetic tree of the PB2 gene full-length sequence of the H9N2 isolates from Kazakhstan.

Trees include four AIV isolates collected in Kazakhstan and selected reference isolates. Full-length sequences with complete open reading frames were used for the phylogenetic analyses, and neighbor-joining trees were generated using MEGA 11. Estimates of the phylogenies were calculated by performing 1000 neighbor-joining bootstrap replicates. The phylogenetic trees of the genes were rooted to A/turkey/Wisconsin/1966 (a-b). Isolates collected from waterfowls are shown in blue, and strain collected from domestic birds is shown in red font.

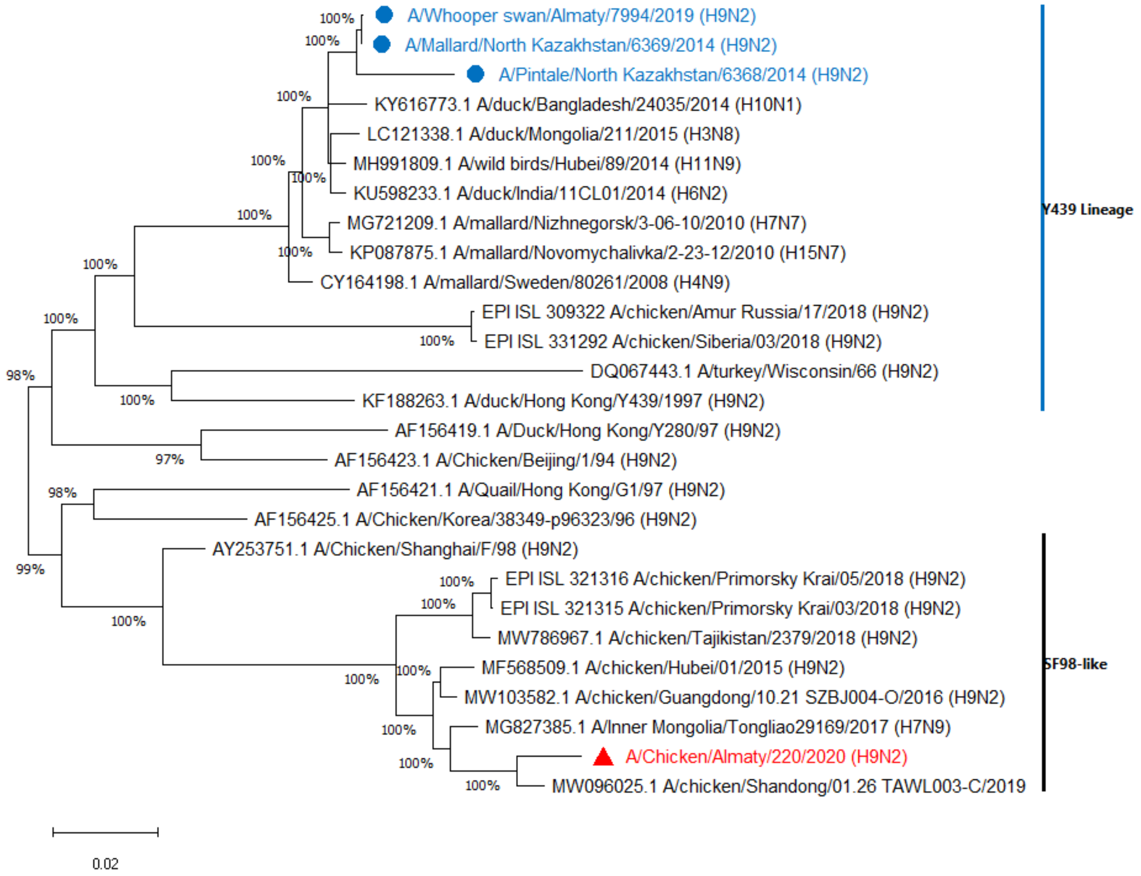

PB1 gene

Supplementary Figure S2. Phylogenetic tree of the PB1 gene full-length sequence of the H9N2 isolates from Kazakhstan.

Trees include four AIV isolates collected in Kazakhstan and selected reference isolates. Full-length sequences with complete open reading frames were used for the phylogenetic analyses, and neighbor-joining trees were generated using MEGA 11. Estimates of the phylogenies were calculated by performing 1000 neighbor-joining bootstrap replicates. The phylogenetic trees of the genes were rooted to A/turkey/Wisconsin/1966 (a-b). Isolates collected from waterfowls are shown in blue, and strain collected from domestic birds is shown in red font.

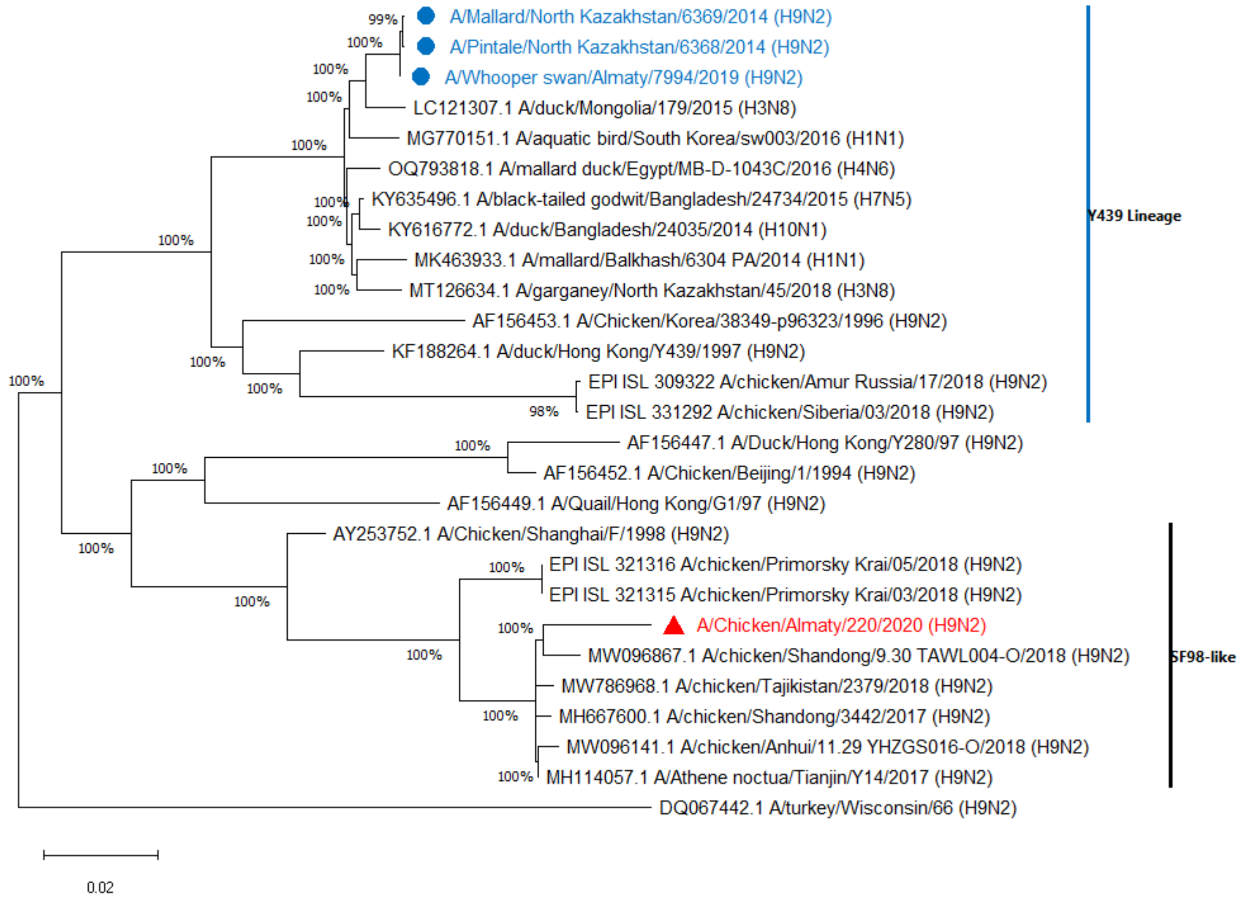

PA gene

Supplementary Figure S3. Phylogenetic tree of the PA gene full-length sequence of the H9N2 isolates from Kazakhstan.

Trees include four AIV isolates collected in Kazakhstan and selected reference isolates. Full-length sequences with complete open reading frames were used for the phylogenetic analyses, and neighbor-joining trees were generated using MEGA 11. Estimates of the phylogenies were calculated by performing 1000 neighbor-joining bootstrap replicates. The phylogenetic trees of the genes were rooted to A/turkey/Wisconsin/1966 (a-b). Isolates collected from waterfowls are shown in blue, and strain collected from domestic birds is shown in red font.

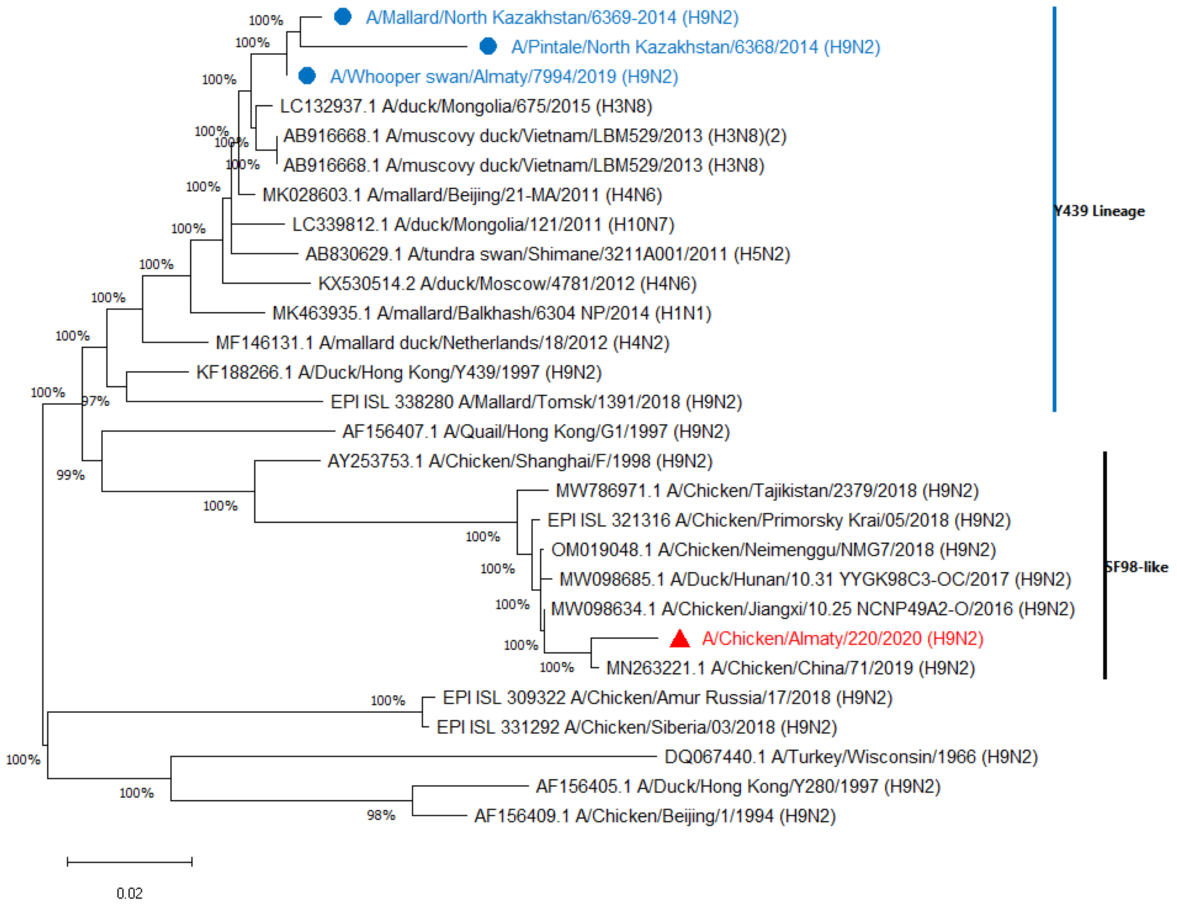

NP gene

Supplementary Figure S4. Phylogenetic tree of the NP gene full-length sequence of the H9N2 isolates from Kazakhstan.

Trees include four AIV isolates collected in Kazakhstan and selected reference isolates. Full-length sequences with complete open reading frames were used for the phylogenetic analyses, and neighbor-joining trees were generated using MEGA 11. Estimates of the phylogenies were calculated by performing 1000 neighbor-joining bootstrap replicates. The phylogenetic trees of the genes were rooted to A/turkey/Wisconsin/1966 (a-b). Isolates collected from waterfowls are shown in blue, and strain collected from domestic birds is shown in red font.

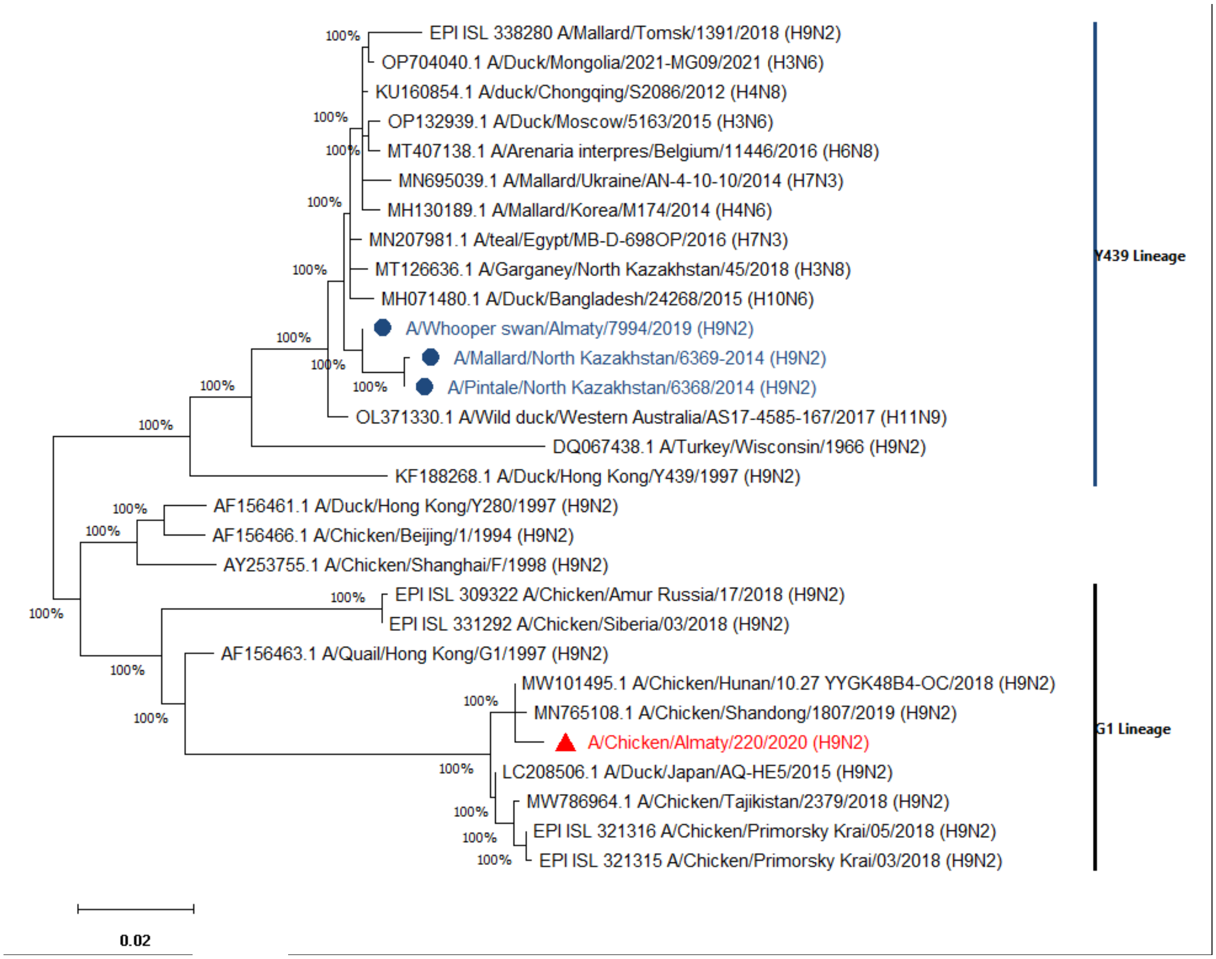

M gene

Supplementary Figure S5. Phylogenetic tree of the M gene full-length sequence of the H9N2 isolates from Kazakhstan.

Trees include four AIV isolates collected in Kazakhstan and selected reference isolates. Full-length sequences with complete open reading frames were used for the phylogenetic analyses, and neighbor-joining trees were generated using MEGA 11. Estimates of the phylogenies were calculated by performing 1000 neighbor-joining bootstrap replicates. The phylogenetic trees of the genes were rooted to A/turkey/Wisconsin/1966 (a-b). Isolates collected from waterfowls are shown in blue, and strain collected from domestic birds is shown in red font.

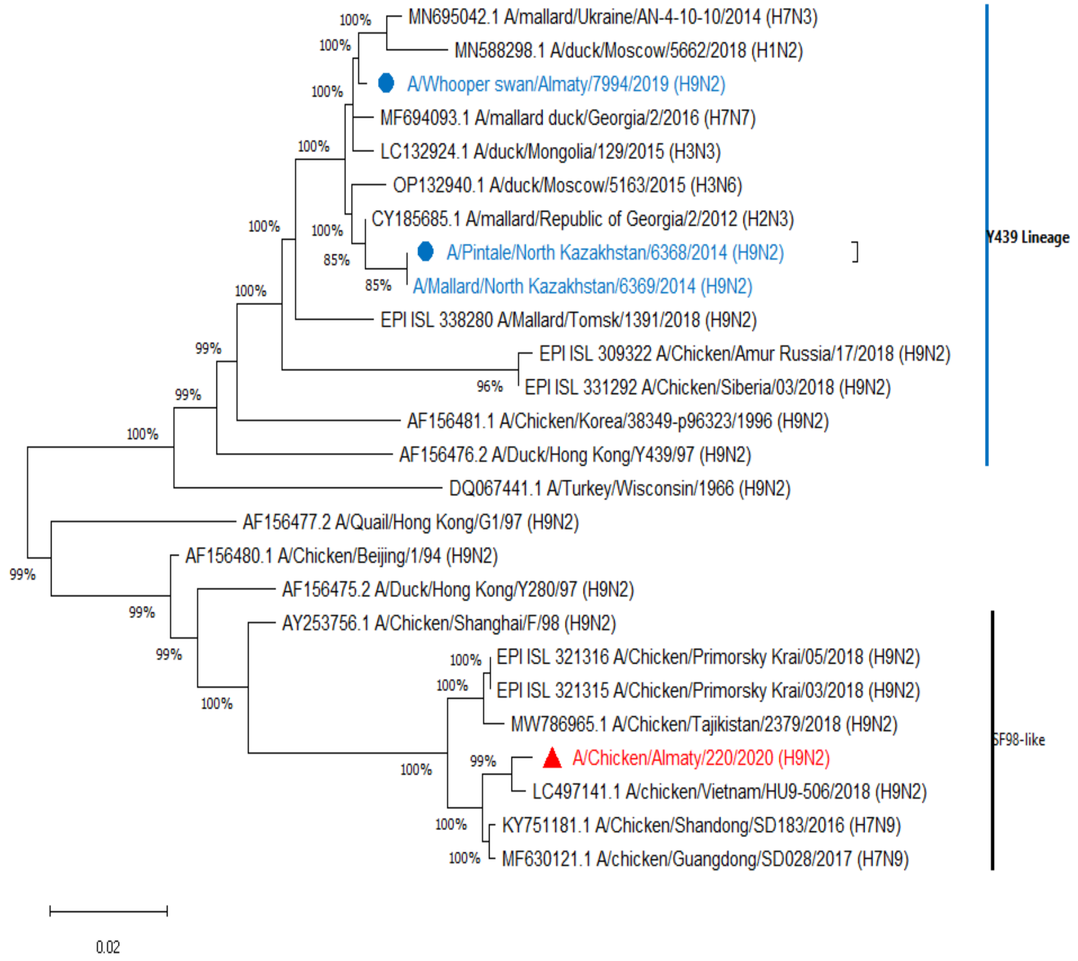

NS gene

Supplementary Figure S6. Phylogenetic tree of the NP gene full-length sequence of the H9N2 isolates from Kazakhstan.

Trees include four AIV isolates collected in Kazakhstan and selected reference isolates. Full-length sequences with complete open reading frames were used for the phylogenetic analyses, and neighbor-joining trees were generated using MEGA 11. Estimates of the phylogenies were calculated by performing 1000 neighbor-joining bootstrap replicates. The phylogenetic trees of the genes were rooted to A/turkey/Wisconsin/1966 (a-b). Isolates collected from waterfowls are shown in blue, and strain collected from domestic birds is shown in red font.
